# Discovery of a Structurally Stable Immunodominant Region in ASFV p30 C-Terminus Using a Panel of Monoclonal Antibodies

**DOI:** 10.1155/tbed/8023572

**Published:** 2025-11-28

**Authors:** Wei Liu, Shandian Gao, Jiaoyan Su, Jinshu Sui, Tong Zhou, Jian Yang, Haiyan Lu, Huichen Guo, Junjun Shao, Huiyun Chang

**Affiliations:** ^1^State Key Laboratory for Animal Disease Control and Prevention, Lanzhou Veterinary Research Institute, Chinese Academy of Agricultural Sciences, Lanzhou, China; ^2^Gansu Province Research Center for Basic Disciplines of Pathogen Biology, Lanzhou Veterinary Research Institute, Chinese Academy of Agricultural Sciences, Lanzhou, China

**Keywords:** African swine fever virus, epitope mapping, immunodominant region, monoclonal antibodies, p30

## Abstract

African swine fever (ASF) is a lethal hemorrhagic viral disease of pigs with devastating global socioeconomic impacts. Due to the absence of a safe and effective vaccine, early surveillance and precise diagnostics are critical for ASF prevention and control. The ASF virus (ASFV) p30 protein, one of the most immunogenic early-expressed antigens during infection, is a prime target for diagnostic assays and subunit vaccines. Its comprehensive antigenic characterization remains crucial for rational vaccine design. In this study, we generated a panel of 20 monoclonal antibodies (MAbs) against the ASFV p30 protein, classifying them into 11 groups based on epitope specificity. MAbs from six groups (subgroups I-1, I-2, and II-1, as well as groups III, IV, and V, collectively comprising 15 MAbs) recognized six distinct epitopes within the C-terminus of p30 (137–194 aa), while MAb 16-5E7E8 (subgroup II-2) recognized a distinct epitope within residues 154–190. The p30 C-terminus (137–194 aa) demonstrated structural stability under denaturing conditions (SDS–PAGE and western blot [WB]) and was identified as an immunodominant region through its reactivity with ASFV-positive sera. Notably, MAbs targeting this immunodominant region exhibited modest neutralizing activity, whereas those binding to other regions showed no neutralizing activity. Furthermore, MAb Q4-1F10B7 (group VI) recognized a linear epitope “^116^TSSFETLFEQ^125^,” MAbs Q1-1G4B2 (group VIII) and Q8-4F9F3 (group IX) recognized conformation-dependent epitopes, and MAb 5-1C8B6 (group VII) likely recognized an aberrant or non-native form of the p30 protein. These results expand the epitope landscape of p30 protein and lay a foundation for ASF diagnosis and vaccine research.

## 1. Introduction

African swine fever (ASF) is a highly contagious, hemorrhagic viral disease affecting domestic swine and wild boars, with significant socioeconomic consequences worldwide. The causative agent, ASF virus (ASFV), is a large, linear double-stranded DNA arbovirus belonging to the only member of the Asfarviridae family, and its genome varies between 170 and 194 kb, encoding up to 68 structural proteins and more than 100 nonstructural proteins [1]. ASFV virions are icosahedral particles with a diameter of around 250 nm, composed of the internal core, the core shell, the inner membrane, the capsid, and the external envelope [2–4].

ASF was first diagnosed in Kenya in 1909 and was described by Montgomery (1921) as a disease different from classical swine fever (CSF) [5]. Subsequently, it spread to other African countries and later reached Western European countries via Spain and Portugal in the 1960s–1980s [5, 6]. By the mid-1990s, ASFV had been largely eradicated in Europe with the exception of Sardinia [5, 7]. However, ASFV reemerged in 2007 in the Caucasus region before rapidly spreading to Eastern European countries [5, 6]. A decade later, a Georgia-07-like genotype II ASFV strain was detected in China in 2018 [8], followed by its further spread to Southeast Asia [9]. Since its emergence in China, ASF has caused severe economic losses to pig husbandry and hindered the trade of pigs and related products. To date, no safe and effective vaccines are available for ASF, making disease control heavily dependent on early detection, strict movement restrictions, and enhanced biosecurity measures [10, 11].

ASFV is currently grouped into 24 genotypes based on the nucleotide sequences of the 3′-end of the B646L gene. However, its virulence of ASFV is not fully associated with the genotypes but rather depends on the specific viral strains and animal species [12, 13]. Initially, the highly virulent genotype II strain was introduced into China, causing acute infections with nearly 100% mortality and persisting for nearly 3 years. Subsequently, low-virulence genotype II variants, which evolved by natural mutations in the genomes of the original highly virulent strains, emerged in 2020 [14]. Furthermore, two additional low-virulence genotype I strains were isolated and identified in China in 2021 [13]. Unlike the highly virulent strains, these attenuated variants caused chronic and persistent infection in pigs, complicating early diagnosis and making disease control more challenging.

Due to the absence of a safe and effective vaccine, reliable laboratory diagnosis and strict biosecurity measures are critically important for the prevention and control of ASF. In acute ASFV outbreaks with high mortality, nucleic acid and antigen detection are crucial, as infected pigs often die before producing detectable antibodies against ASFV. Conversely, in cases of infection with lower virulent variants that cause chronic and persistent disease, the detection of ASFV-specific antibodies provides a viable and efficient diagnostic procedure for conducting ASF surveillance and confirming the ASFV-infected herds.

The p30 protein, encoded by the CP204L gene, is highly expressed during the early stage of infection and can be detected as early as 2 h postinfection in ASFV-infected macrophages, persisting throughout the infection cycle [15]. To identify host proteins targeted by p30, Hernaez et al. [16] employed a nuclear yeast two-hybrid system to identify an interaction between p30 and heterogeneous nuclear ribonucleoprotein K (hnRNP-K), suggesting a potential role for p30 in the downregulation of host mRNA translation following ASFV infection. Similarly, using the DUAL membrane yeast two-hybrid system, Chen et al. [17] identified seven cellular proteins interacting with p30, which may be involved in the endocytic pathway and the innate immune response. Nevertheless, the precise mechanism of viral entry remains to be defined. More recently, Dolata et al. [18] reported that p30 interacts with the homotypic fusion and vacuole protein sorting (HOPS) protein VPS39, which modulates endosomal trafficking and promotes lysosome clustering during virus infection. Collectively, these studies indicate that p30 is a multifunctional protein participating in multiple stages of ASFV life cycle, including viral entry, intracellular trafficking, and the modulation of host gene expression and immune responses. Notably, Gómez-puertas et al. [19] demonstrated that antibodies against p30 are able to inhibit virus internalization in porcine macrophages and Vero cells [19]. Consequently, p30 was often designed in vaccine studies together with other structural proteins. In addition to its early expression and potent immunogenicity, p30 demonstrates strong antigenicity in infected hosts [19–21]. Supporting this, anti-p30 antibodies have been detected in sera and oral fluid samples from inoculated pigs as early as 6 days postinoculation (dpi) and 8 dpi, respectively. Furthermore, by 12 dpi, antibody levels against p30 significantly surpassed those detected against p54 and p72 [10]. These properties make p30 an ideal diagnostic target for early detection of ASF.

Epitope identification is pivotal for the rational design of subunit vaccines and the development of diagnostic assays. Although several antigenic epitopes of p30 have been identified in previous studies (summarized in Table 1), the specific immunodominant region within its C-terminal domain has remained uncharacterized. Here, we provide the first systematic dissection of this critical region.

In this study, we generated 20 monoclonal antibodies (MAbs) against the ASFV p30 protein. Through chemiluminescence immunoassay (CLIA) additivity tests and epitope mapping, these MAbs were classified into 11 distinct groups. Among these, seven groups (comprising 16 MAbs) recognized epitopes within the C-terminal region (137–194 aa). In contrast, the linear epitope “^116^TSSFETLFEQ^125^” was precisely identified by MAb Q4-1F10B7, whereas MAbs Q1-1G4B2 and Q8-4F9F3 recognized conformation-dependent epitopes. Notably, MAb 5-1C8B6 exclusively recognized an aberrant or non-native p30 conformation. Additionally, a structurally stable immunodominant region (137–194 aa) was first identified through western blot (WB) and enzyme-linked immunosorbent assay (ELISA) analysis, demonstrating significantly stronger reactivity with ASFV-positive sera than other p30 fragments or previously reported epitopes. These results provide critical insights for developing ASFV diagnostics and subunit vaccines.

## 2. Materials and Methods

### 2.1. Virus, Cell, and Sera

The ASFV/II/SC/2019 strain was preserved in the biosafety level 3 (BSL-3) laboratory of Lanzhou Veterinary Research Institute (LVRI), Chinese Academy of Agricultural Science (CAAS). Primary porcine alveolar macrophages (PAMs) were collected from the lungs of healthy pigs (~25 kg) using bronchoalveolar lavage according to the method described by Carrascosa et al. [29]. The PAMs were resuspended with cold FBS supplemented with 10% dimethyl sulfoxide and frozen at a 1°C/min constant cooling rate before storing in liquid nitrogen. One ASFV-positive serum was purchased from the National Center for Veterinary Culture Collection (CVCC), and four ASFV-positive sera were collected and stored in our laboratory, including P3352, P3421, P3422, and P3423. SP2/0 cells were stored in our laboratory.

### 2.2. Expression and Purification of p30 Protein

The ASFV CP204L gene sequence from strain SY18 (GenBank: MH766894.1) was codon-optimized, synthesized, and cloned into pET-28a vector by *Eco*RI and *SalI* restriction sites. The recombinant plasmid was transformed into *Escherichia coli* BL21(DE3) and induced with 1 mM isopropyl-*β*-D-thiogalactoside (IPTG) for 8 h at 37°C in LB medium. Pellets of bacterial cells were harvested by centrifugation at 5000 g for 5 min, and then the target protein was purified using the previously described method [30]. Then, the purified protein was refolded by gradually reducing the concentration of urea and imidazole and finally exchanged into tris buffer (20 mM tris, 250 mM NaCl, 10% glycerol) [31].

### 2.3. MAb Production

The 8-week-old BALB/c mice, purchased from the Laboratory Animal Center of LVRI, were injected intramuscularly with 0.1 mL of p30 renatured protein (200 μg/mL) emulsified with Freund's complete adjuvant at a 1:1 ratio (v/v). After 21 days of first immunization, the mice were boosted with p30 renatured protein emulsified in equal proportions with Freund's incomplete adjuvant three times with 2-week intervals. Three days prior to harvesting the spleen, the mice were injected intraperitoneally with 20 μg p30 renatured protein. Splenocytes isolated from two mice were fuzed with SP2/0 cells. Hybridomas were cultured in Dulbecco's modified Eagle medium (DMEM; BI) containing hypoxanthine–aminopterin–thymidin (HAT; Sigma–Aldrich, USA) supplement and 20% fetal bovine serum (FBS; BI, China) at 37°C under 5% CO_2_ atmosphere. Hybridomas were screened by indirect ELISA for positive clones, which were then subjected to three rounds of subcloning by limiting dilution. The selected hybridomas were injected into the abdominal cavities of 12-week-old BALB/c mice to obtain ascites according to Miao et al. [30]. The ascites were collected and purified using a 1-mL HiTrap Protein G HP column connected to an ÄKTA pure instrument (GE Healthcare, USA) according to the manufacturer's instructions. The subclass specificity of MAbs was identified by the SBA Clonotyping System-HRP Kit (SouthernBiotech, USA).

### 2.4. Indirect Immunofluorescence Assay (IFA)

PAMs monolayers in 12-well plates cultured with RPMI-1640 supplemented with 15% FBS were infected with ASFV/II/SC/2019 at a multiplicity of infection (MOI) of 0.1 for 48 h. At the end of infection, the media from the wells were removed, and cells were fixed with 4% paraformaldehyde for 30 min and permeabilized with 0.1% Triton X-100 for 10 min. After blocking with 2% BSA for 1 h, the hybridoma supernatant was diluted at a 1:20 ratio, added to wells, and incubated for 1 h. The cells were then incubated with the tetramethyl Rhodamine isothiocyanate (TRITC)-conjugated goat anti-mouse IgG (Abcam, UK) at a dilution of 1:1000. Nuclei were stained with DAPI, and the plates were examined with a fluorescence microscope (Leica Microsystems, Germany).

### 2.5. Expression of the Fusion Protein

To map the epitope recognized by each MAb, we designed a series of overlapping peptides, truncated peptides, and sequential alanine-scanning peptides. The coding nucleotide sequences were codon-optimized, chemically synthesized, and cloned into the pGEX-4 T-1 vector using *BamHI* and *XhoI* restriction sites. The recombinant plasmid was then transformed into *E. coli* BL21 (DE3). Following induction, the recombinant proteins were expressed in the bacterial cells and purified by glutathione S-transferase (GST) affinity chromatography, and subsequently analyzed by WB and indirect ELISA.

### 2.6. WB

Each fusion protein was mixed with 4x loading buffer, heated, and separated by SDS–PAGE on a 10%–15% tris-glycine gel, with ~100 ng loaded per lane. Proteins were then transferred to a polyvinylidene difluoride (PVDF) membrane (Bio-Rad, USA), followed by blocking with 5% skim milk in PBST. The membrane was probed overnight at 4°C with hybridoma supernatant (1:20 dilution in PBST). After washing five times with PBST, the membrane was incubated for 1 h at room temperature with HRP-conjugated goat anti-mouse IgG (Sigma, USA; 1:10,000 dilution in PBST). After washing, proteins were detected using chemiluminescence (CL) reagents and visualized with an imaging system (LI-COR, NE, USA).

### 2.7. Indirect CLIA and Indirect ELISA

Purified proteins were diluted in carbonate-bicarbonate buffer (pH 9.6) and coated onto 96-well plates (white plates for CLIA or polystyrene plates for ELISA) at 100 μL/well, followed by overnight incubation at 4°C. After washing five times with PBST, plates were blocked with blocking buffer (2% BSA in PBST) at 37°C for 2 h. The hybridoma supernatant was then diluted, added to the plate, and incubated at 37°C for 30 min. After washing, HRP-conjugated goat anti-mouse IgG was added and incubated at 37°C for 30 min. For indirect CLIA, 100 μL of CL substrate (Key-Bio, China) was added, and after a 5-min reaction, CL signals were measured using a Varioskan Lux instrument (Thermo Fisher Scientific, USA). For indirect ELISA, 50 μL of TMB was added and incubated for 10–15 min at 37°C. The reaction was stopped by adding 50 μL/well 2M H_2_SO_4_, and the absorbance at 450 nm was measured using a Varioskan Lux instrument.

### 2.8. CLIA Additivity Test

To preliminarily determine whether the 20 MAbs recognized distinct epitopes, we developed a modified CLIA-based additivity test instead of the conventional ELISA [32, 33]. Briefly, the supernatant from the first hybridoma was serially diluted and added to the plate, which had been precoated with the purified p30 protein (0.125 μg/mL in 100 μL per well), followed by a 30-min incubation at 37°C. After washing, the plate was incubated with the secondary hybridoma supernatant under the same conditions. Subsequent steps followed the indirect CLIA protocol described above.

For quantitative analysis, an additivity index (AI) was defined for each antibody pair according to the formula as follows:

  $$\begin{array}{c} \text{AI}=\left(2\times A_{1+2}/\left(A_{1}+A_{2}\right)-1\right)\times 100, \end{array}$$

where *A*_1_, *A*_2_, and *A*_1 + 2_ represent the CL values of the first MAb alone (*A*_1_), the second MAb alone (*A*_2_), and their combination (*A*_1 + 2_), respectively. Theoretically, when the first MAb saturates the antigen's epitope, a second MAb recognizing the same epitope will be unable to bind, preventing any increase in CL values and driving the AI toward zero. Conversely, if the two antibodies bind to distinct, nonoverlapping sites, the addition of the second MAb will enhance CL values, resulting in a higher AI. A cut-off value of AI at 50% is typically used to distinguish these binding patterns.

### 2.9. Sequence Comparisons

To evaluate the conservation of epitopes recognized by the MAbs from 11 groups among different ASFV strains, 17 representative p30 sequences, selected from 252 ASFV complete genomes, were aligned and analyzed using the BioEdit software (version 7.2.5).

### 2.10. Virus Neutralization Assay

The MAbs were grouped into three representative combinations (2-1B3G3 and 7-1C11F4, Q3-1C9F6 and 16-5E7E8, Q1-1G4B2 and Q8-4F9F3) based on epitope similarity to initially assess their neutralizing potential against the ASFV/II/SC/2019 strain according to a previously established method [30]. MAb 8E2, which recognizes the FMDV G-H loop, served as the negative control. Briefly, MAbs were serially diluted two-fold from 200 μg/mL to 12.5 μg/mL in RPMI-1640 medium using 96-well cell culture plates, with a total volume of 50 μL per well. Subsequently, 100 HAD_50_ of ASFV in 50 μL of RPMI-1640 medium was added to each well. Each antibody dilution was tested in duplicate. After incubation for 4 h at 37°C, about 5 × 10^4^ PAM cells in 50 μL of RPMI-1640 medium containing 15% FBS were added to each well. After incubation in an atmosphere of 5% CO_2_ at 37°C for 36 h, the ASFV genomes were extracted from each well using Steadypure virus DNA/RNA extraction kit (AG, China) and quantified by qPCR (Pro Taq HS Premix Probe kit; AG, China).

The recombinant plasmid p72-pET-28a was constructed and 10-fold serially diluted to serve as a template for qPCR amplification. A standard curve was established through linear regression analysis between the logarithmic value of the recombinant plasmid copy numbers and the Ct value. The copy number of the p72 gene in each sample was calculated according to its Ct value and the established standard curve. The virus neutralization rate (%) was calculated as follows:

  $$\begin{array}{c} \text{Neutralization rate }\left(\%\right)=100\%-\frac{\text{copy number of }p72\text{ gene in sample}}{\text{copy number of p}72\text{ gene in negative control }\left(\text{MAb}8E2\right)}\%. \end{array}$$

## 3. Results

### 3.1. Production and Characterization of MAbs Against ASFV p30

A total of 20 hybridoma cells secreting anti-p30 MAbs were obtained by screening with an indirect ELISA, using purified recombinant p30 protein (Figure S1) coated at a concentration of 1 μg/mL in a volume of 100 μL per well. Subsequently, IFA revealed that 13 of the 20 MAbs exhibited reactivity with ASFV-infected PAMs, while seven MAbs (12-3B2G5, 17-5C11E3, Q3-1C9F6, 16-5E7E8, 8-2B4A3, 15-4B9H10, and 5-1C8B6) showed no detectable reactivity (Table 2, Figure S2). WB analysis demonstrated that three MAbs (Q1-1G4B2, Q8-4F9F3, and 5-1C8B6) failed to recognize ASFV-infected PAM lysates, with an additional three (1-1D2B3, 8-2B4A3, and 15-4B9H10) displaying only weak reactivity (Table 2, Figure S3). The reactivity of these MAbs with the p30 protein was also further confirmed by WB. All MAbs except Q1-1G4B2 and Q8-4F9F3 reacted with the recombinant p30 protein (Table 2, Figure S4). Both IFA and WB analyses demonstrated that MAbs Q1-1G4B2 and Q8-4F9F3 recognized conformation-dependent epitopes, whereas MAb 5-1C8B6 likely recognized an aberrant p30 form expressed in the prokaryotic expression system.

### 3.2. CLIA Additivity Test

To classify the MAbs through the CLIA additivity test, it was critical to first confirm antigen saturation by each individual MAb. Accordingly, antigen saturation curves were established for each MAb using indirect CLIA (Figure S5). For the CLIA additivity test, antibody dilutions were selected when each MAb reached near-saturation (defined as <35% difference in CL values between adjacent hybridoma supernatant dilutions), while ensuring comparable CL values between paired MAbs. CLIA additivity test indicated that the 20 MAbs were classified into nine distinct groups (groups I–IX), as shown in Figure S6 and Table 2.

### 3.3. Mapping of the Epitopes

**Figure 1:**
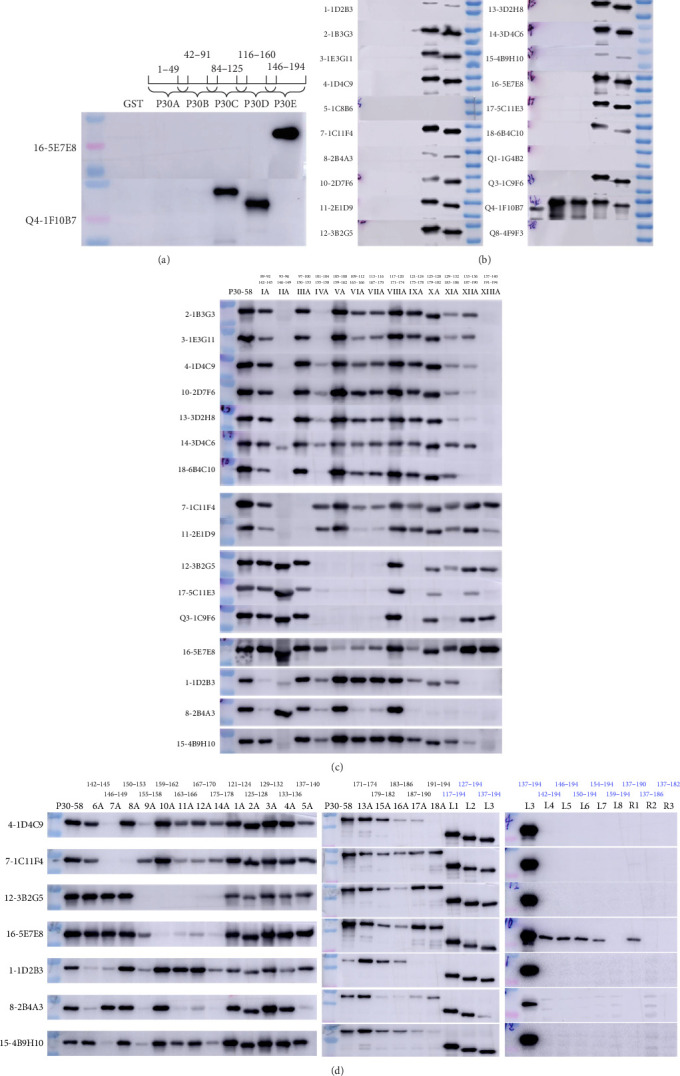
Mapping of epitopes recognized by 20 MAbs. (A) Five overlapping peptides (p30-A, 1−51 aa; p30-B, 41−93 aa; p30-C, 82−127 aa; p30-D, 114−162 aa; and p30-E, 144−194 aa) were expressed and analyzed by WB. (B) Five ultra-long overlapping peptide fragments (p30-15, 1−124 aa; p30-26, 23−146 aa; p30-37, 45−168 aa; p30-48, 67−194 aa; and p30-58, 89−194 aa) were used for WB to analyze their reaction with 20 MAbs. (C) A series of eight alanine-substituted recombinant proteins (IA−XIIIA) was analyzed with the remaining 16 MAbs, and the number at the top of IA−XIIIA represents the alanine-substituted location in every recombinant protein. (D) Four sequential alanine-substituted proteins (1A−18A), N-terminal (L1−L8) and C-terminal truncated peptides (R1−R3) were analyzed with MAbs from seven groups. The number at the top of 1A−18A represents the alanine-substituted location in every recombinant protein, and the number at the top of L1−L8 and R1−R3 (blue font) represents the amino acid length of truncated peptides.

The primary epitope mapping was carried out by WB using five overlapping peptides (p30-A, -B, -C, -D, -E). The results showed that only 16-5E7E8 reacted with p30-E (146–194 aa), while Q4-1F10B7 reacted with both p30-C (84–125 aa) and p30-D (116–160 aa). However, none of the remaining 18 MAbs showed reactivity with any of these five overlapping peptides. The shared overlapping sequence (^116^TSSFETLFEQ^125^) between p30-C and p30-D defines the minimal epitope recognized by the MAb Q4-1F10B7 (Figure 1A). Indirect ELISA was also performed using purified p30-A, -B, -C, -D, -E as coating antigens, and results were consistent with those obtained from WB analysis (data not shown).

**Figure 2:**
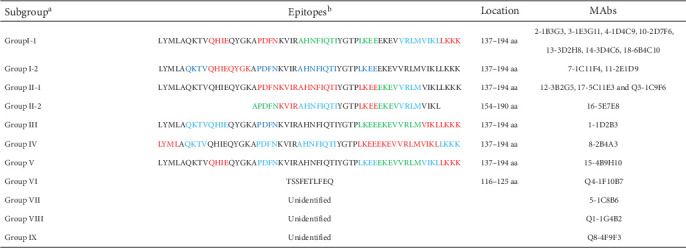
Profile of the fine epitopes recognized by 20 MAbs and their critical residues. Note: ^a^20 MAbs were classified into 11 groups based on WB and CLIA additivity test. ^b^In epitope mapping diagram, the color gradient of red, blue, and green represents progressively decreasing criticality of amino acid residues for antibody binding. Red-colored residues indicate complete abolition of antibody-epitope interaction upon mutation, blue denotes residues where alanine substitution causes significant binding reduction, while green marks position where alanine substitution lead to only mild binding reduction.

To further identify the epitopes recognized by MAbs, five ultra-long overlapping peptide fragments (p30-15, p30-26, p30-37, p30-48, p30-58) were expressed and analyzed by WB. The results showed that all MAbs except 5-1C8B6, Q1-1G4B2, and Q8-4F9F3 reacted with both p30-48 and p30-58 (Figure 1B), which further confirmed that Q1-1G4B2 and Q8-4F9F3 recognized conformation-dependent epitopes. Subsequent analysis utilizing a series of eight alanine-substituted recombinant proteins (IA to XIIIA) classified the remaining 16 MAbs into seven distinct groups. The MAbs within group I were further classified into subgroup I-1 (2-1B3G3, 3-1E3G11, 4-1D4C9, 10-2D7F6, 13-3D2H8, 14-3D4C6, and 18-6B4C10) and subgroup I-2 (7-1C11F4, 11-2E1D9), while those within group II were divided into subgroup II-1 (12-3B2G5, 17-5C11E3, and Q3-1C9F6) and subgroup II-2 (16-5E7E8). The classification of group III (1-1D2B3), IV (8-2B4A3), and V (15-4B9H10) in the additivity test was consistent with the results of alanine-scanning analysis (Figures 1C and 2).

To precisely map the epitopes and identify the critical amino acid residues, a panel of four sequential alanine-substituted proteins, along with N-terminal and C-terminal truncated peptides, was expressed and analyzed by WB. The results revealed that MAb from subgroup II-2 recognized the L7 (154–194 aa) and R1 (137–190 aa), indicating that the minimal epitope is located within 154–190 aa, while MAbs in subgroups I-1, I-2, and II-1 as well as groups III, IV, and V recognized an epitope region spanning 137–194 aa (Figures 1D and 2). Notably, MAbs in subgroup I-1 and I-2 shared recognition of similar critical amino acid residues, while those in the subgroup II-1 and II-2 recognized another set of similar critical residues (Figures 1D and 2). These findings explain the AI values <50% in CLIA additivity test when adding MAbs from a different subgroup that target similar critical residues.

### 3.4. The Stability of the p30 C-Terminus

**Figure 3:**
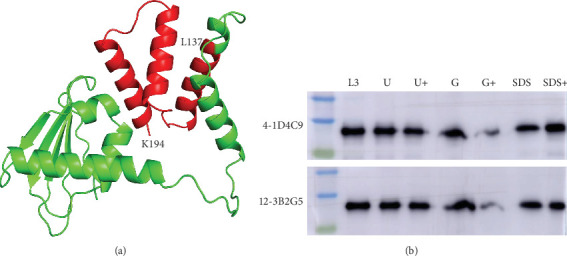
Structure features and stability of the p30 C-terminal region. (A) Predicted structure of the p30 protein generated by AlphaFold 2. (B) Reaction of MAbs 4-1D4C9 and 12-3B2G5 with the L3 under various denaturing conditions. The bacterial cells expressing recombinant L3 were lysed by ultrasonication in PBS alone or in PBS containing 6M urea, 6M guanidinium chloride, or 5% SDS. The lysates were mixed with 4x loading buffer and either boiled for 10 min or left unboiled before separation by SDS-PAGE and transfer to a PVDF membrane. Immunoblotting was performed using MAbs 4-1D4C9 and 12-3B2G5 as primary antibodies. Lane designations were as follows: L3: lysed in PBS, boiled; U: treated with 6M urea, unboiled; U+: treated with 6M urea and boiled; G: treated with 6M guanidinium chloride, unboiled; G+: treated with 6M guanidinium chloride and boiled; SDS: treated with 5% SDS, unboiled; and SDS+: treated with 5% SDS and boiled.

Twenty MAbs were classified into 11 groups based on their epitope binding profiles. Notably, seven of these groups (I-1, I-2, II-1, II-2, III, IV, and V) recognized epitopes located at the p30 C-terminus, each exceeding 15 amino acids in length. Strikingly, these epitopes remained detectable under denaturing conditions (SDS, boiling, and methanol treatment) during SDS–PAGE and WB analysis. The reason may relate to the structure of p30 C-terminus, which is composed of three *α*-helix (Figure 3A). Further validation revealed that the *N*-terminal truncated protein L3 (137–194 aa) remained reactive with MAbs 4-1D4C9 and 12-3B2G5 under denaturing treatments (6M urea, 6M guanidinium chloride, 5% SDS, and boiling), with only the combined treatment with 6M guanidinium chloride and boiling resulting in reduced binding (Figure 3B). Collectively, these results demonstrated that the p30 C-terminus adopts a stable or flexibly robust conformation that withstands denaturation, preserving its antigenic integrity.

### 3.5. The Immunodominant Region of p30

**Figure 4:**
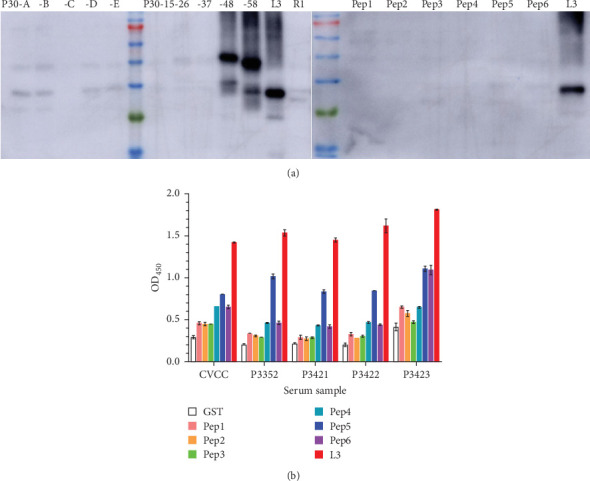
The reactivity of p30-A–p30-E, p30-15, p30-26, p30-37, p30-48, p30-58, L3, R1 and previously reported epitopes (Pep1–Pep6) with ASFV-positive sera. (A) Pep1–Pep6 were expressed and analyzed with ASFV-positive serum (P3352, stored in our laboratory) by WB. (B) Indirect ELISA analysis of the reactivity of GST, Pep1–Pep6, and L3 with five ASFV-positive sera. The sera included four in-house samples (P3352, P3421, P3422, P3423) and one commercial sample from the National Center for Veterinary Culture Collection (CVCC). Pep1 is ^84^NMILHVLF^91^ [11], Pep2 is ^96^ESSASSENIH^105^ [11], Pep3 is ^116^TSSFETLFEQ^125^ [11, 28], Pep4 is ^146^QHIEQYGKAPDFNKV^160^ [11], Pep5 is ^61^DIVKSARIYAGQGYTEHQAQEEWNMILHVLFEE^93^ [24], and Pep6 is ^12^EVIFKTDLRSSSQ^24^ [25].

The reactivity of various fragments (p30-A to p30-E, p30-15, p30-26, p30-37, p30-48, p30-58, L3, R1) and previously reported epitopes (Pep1 to Pep6) [11, 24, 25, 28] was assessed against ASFV-positive sera. Strong reactivity was observed for the p30 C terminus (p30-48, 67–194 aa; p30-58, 89–194 aa; and L3, 137–194 aa), with the intensity of reaction correlating with C-terminus fragment length (Figure 4A). In contrast, Pep1 to Pep6 exhibited significantly weaker reactivity compared to L3 (Figure 4A,B), suggesting that L3 represents the immunodominant region of p30.

### 3.6. The Conservation of Epitopes Among Different ASFV Isolates

**Figure 5:**
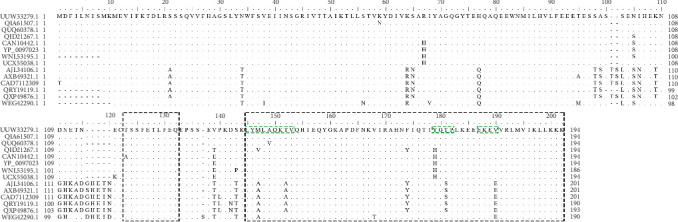
Sequence alignment of p30 from 17 ASFV representative sequences. The dots indicate the same amino acid, and no acid amino is denoted with a dashed line.

The conservation of epitopes recognized by MAbs from eight groups was evaluated across different ASFV strains. Sequence alignment revealed that the epitope recognized by group VI MAb was highly conserved, exhibiting only a single amino acid substitution (Thr^116^–Ala^116^). Similarly, epitopes recognized by MAbs from subgroups I-1, I-2, II-1, and II-2, as well as groups III, IV, and V, showed relatively high conservation, with minor polymorphisms occurring at positions Met^139^, Thr^144^, Tyr^171^, Thr^173^, and Val^182^ in some isolates. Importantly, these variations were noncritical and did not significantly compromise MAb reactivity (Figure 5).

### 3.7. Virus Neutralization

**Figure 6:**
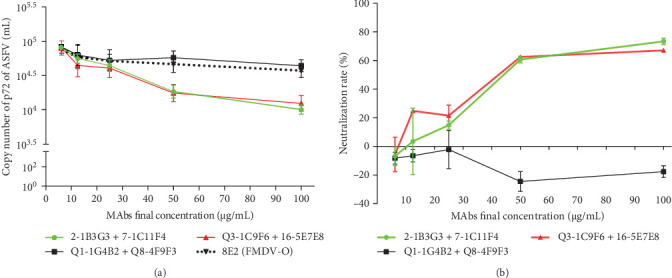
The qPCR neutralization assay. (A) The copy numbers of the p72 gene were calculated in MAbs from 6 groups. The indicated concentration of MAb combinations, including 2-1B3G3 (subgroup I-1) and 7-1C11F4 (subgroup I-2), Q3-1C9F6 (subgroup II-1) and 16-5E7E8 (subgroup II-2), Q1-1G4B2 (group VIII) and Q8-4F9F3 (group IV), as well as MAb 8E2 recognizing the FMDV G-H loop, was mixed with ASFV/II/SC/2019 (100 HAD50). After incubation for 4 h at 37°C, the mixture was inoculated into PAMs and cultured for 36 h. Then, viral DNA was extracted and quantified by qPCR. (B) The virus neutralization rate (%) was calculated.

To assess the neutralization capability of MAbs, qPCR-based neutralization assays were performed. The standard curve of logarithmic value of p72 gene copy number and Ct value was established, and the linear regression equation is: *Y* = −3.238*X* + 42.93 (*R*^2^ = 0.9995; Figure S7). The p72 gene copy numbers of each sample were then calculated according to the established linear equation and their respective Ct values (Figure 6A). The neutralization assays revealed that MAb combinations of 2-1B3G3 and 7-1C11F4, as well as Q3-1C9F6 and 16-5E7E8, demonstrated dose-dependent neutralization activity against ASFV. At 50 μg/mL, these combinations achieved a neutralization rate of 60.6% and 62.5%, respectively, while at 100 μg/mL, the neutralization rate increased to 73.4% and 67.0%. In contrast, the combination of Q1-1G4B2 and Q8-4F9F3, which recognized a conformation-dependent epitope, exhibited no detectable neutralizing activity against ASFV (Figure 6B).

## 4. Discussion

Epitope identification helps researchers to gain deeper insights into antigenic structure, immunological functions, and virus–antibody interaction mechanisms. It also provides critical information for the rational design of subunit vaccines and the development of diagnostic assays [11, 30]. In this study, a panel of 20 MAbs against the ASFV recombinant p30 protein was obtained. Epitope mapping for these MAbs was performed using CLIA additivity test and WB with five overlapping peptides, five ultra-long overlapping peptide fragments, a series of sequential alanine-substituted proteins, and N-terminal and C-terminal truncated peptides. The results demonstrated that these MAbs were classified into 11 distinct groups based on their epitope specificity (Figure S6 and Figure 2).

Subsequent epitope mapping analysis classified 15 MAbs into three subgroups (I-1, I-2, and II-1) and three groups (III, IV, and V), each recognizing distinct epitopes within residues 137–194. MAb 16-5E7E8 (subgroup II-2) specifically recognized an epitope localized to residues 154–190 (Figure 2). Notably, MAbs in the subgroups I-1 and I-2 shared some critical residues, while those in the subgroups II-1 and II-2 shared another set of some critical residues. This accounted for indistinguishable profiles of CLIA additivity between subgroups I-1 and I-2 or subgroups II-1 and II-2. Strikingly, unlike the conventional linear epitopes previously reported [11, 25, 27], all MAbs from the seven groups in our study recognized extended epitopes (> 15 aa) that maintained antigenicity under denaturing conditions (SDS–PAGE and WB), suggesting remarkable structural robustness in the p30 C-terminal region. This finding aligned with previous work by Petrovan et al. [24], which attributed such behavior to an intrinsically disordered region spanning residues 91–143. Furthermore, although Wu et al. [11] reported that seven MAbs reacted with larger p30 fragments in ELISA and WB, they did not identify the precise epitope, characterize its structural stability, or demonstrate its immunodominance. Intriguingly, while residues 137–145 were not directly involved in MAbs binding, their deletion resulted in the abolishment of reactivity with the corresponding MAbs (Figure 1D), suggesting their essential role in maintaining the structural integrity of the p30 C-terminus. Further structural validation via cryo-electron microscopy is warranted to elucidate these intriguing phenomena. Furthermore, the region spanning 137–194 aa constituted an immunodominant region of p30. It showed significantly stronger reactivity with ASFV-positive sera than other p30 fragments and previously reported epitopes (Figure 4). Sequence alignment analysis revealed remarkable evolutionary conservation of the immunodominant region across diverse ASFV isolates, with mutations being confined to noncritical residues (Figure 5). These characteristics highlight its critical roles in the development of subunit vaccines and the improvement of diagnostic assay sensitivity. Additionally, the linear epitope “^116^TSSFETLFEQ^125^,” recognized by MAbs Q4-1F10B7 (group VI), matches previously reported findings [11, 28]. Meanwhile, MAbs Q1-1G4B2 (group VIII) and Q8-4F9F3 (group IX) exhibited reactivity with ASFV-infected PAMs in IFA analysis and recombinant p30 protein in ELISA, but failed to recognize ASFV or p30 protein in WB, indicating their recognition of conformation-dependent epitopes. In contrast, MAb 5-1C8B6 (group VII) reacted exclusively with recombinant p30 protein but not ASFV in IFA or WB, suggesting its preferential specificity for an aberrant or non-native form of p30 protein expressed in the prokaryotic expression system.

ASFV p30 is widely recognized as a highly immunogenic protein capable of eliciting robust antibodies during infection [15, 21], and antibodies against p30 have been implicated in the inhibition of ASFV internalization [19]. In our study, a qPCR-based neutralization assay demonstrated limited neutralizing activity in MAbs targeting the p30 C-terminus (combinations of subgroups I-1 and I-2, as well as subgroups II-1 and II-2). This observation aligned with prior evidence demonstrating that immunization with alphavirus replicon particles expressing p30 (RP-30) induced low levels of neutralizing activity in swine [23]. The low neutralizing activity may be attributed to the complex multilayered structures of ASFV and its multiple entry mechanisms, including receptor-mediated endocytosis, clathrin-mediated endocytosis, macropinocytosis, and phagocytosis [34]. Notably, MAbs Q1-1G4B2 and Q8-4F9F3, which recognize conformation-dependent epitopes, exhibited undetectable neutralizing activity against ASFV.

Furthermore, compared to the ELISA additivity test (Figure S8, Table S1), the CLIA additivity test employed in this study demonstrated superior MAb screening efficacy. This method enhances the discrimination of MAbs targeting distinct epitopes, thereby minimizing redundant antibody production. The enhanced performance is attributable to CLIA's broad linear dynamic range.

## 5. Conclusion

In this study, we generated a panel of 20 MAbs against ASFV p30. Epitope mapping revealed that 16 of these MAbs recognized a structurally stable, immunodominant region (137–194 aa) containing at least seven distinct epitopes. Of these, MAbs from both subgroup pairs (I-1 and I-2, as well as II-1 and II-2) exhibited modest neutralizing activity in the qPCR-based neutralization assay. Furthermore, MAb Q4-1F10B7 specifically recognized the linear epitope “^116^TSSFETLFEQ^125^,” while MAbs Q1-1G4B2 and Q8-4F9F3 recognized conformation-dependent epitopes. Notably, MAb 5-1C8B6 appeared to target an aberrant or non-native form of p30 protein. These results expand the known epitope landscape of p30 and provide valuable insights that advance both the development of diagnostic methods and the design of subunit vaccines for ASFV.

## Figures and Tables

**Table 1:** The epitopes of ASFV p30 demonstrated in previous research.

Epitopes	Location	References
LKEEEKEVVRLMVIKLLKKNKL	175–196	[22]
ETNECT**SSFETLFEQ**EPSSE	111–130	[23]
DIVKSAHIYAGQGYTEHQAQEEWNMILHVLFEE	61–93	[24]
NMILHVLF	84–91	[11]
ESSASSENIH	96–105	
T**SSFETLFEQ**	116–125	
QHIEQYGKAPDFNKV	146–160	
TVQHIEQYGKA	144–154	[25]
EVIFKTD	12–18	
KTDLRSSSQVVFHAGSLYNWFSVEIINSGRIVT	16–48	[26]
LFEQEPS	122–128	
HNFIQTI	164–170	[27]
NECT**SSFETLFE**	113–124	[21]
VKYDIVKSARIYAGQGY	58–74	[20]
**SSFETLFEQ**	117–125	[28]
The C-terminus of p30		[11, 24]

*Note:* The epitope “SSFETLFEQ” has been widely documented across multiple studies.

**Table 2:** Reactivity of 20 MAbs and the classification based on CLIA additivity test.

MAbs	Groups^a^	Light chains	MAbs subclass	IFA^b^	WB		ELISA^c^
					The reaction with ASFV^b^	The reaction with p30 protein^c^	
2-1B3G3	Group I	*k*	IgG1	+	+	+	+
3-1E3G11	Group I	*k*	IgG1	+	+	+	+
4-1D4C9	Group I	*k*	IgG2a	+	+	+	+
10-2D7F6	Group I	*k*	IgG2a	+	+	+	+
13-3D2H8	Group I	*k*	IgG1	+	+	+	+
14-3D4C6	Group I	*k*	IgG1	+	+	+	+
18-6B4C10	Group I	*k*	IgG1	+	+	+	+
7-1C11F4	Group I	*k*	IgG1	+	+	+	+
11-2E1D9	Group I	*k*	IgG1	+	+	+	+
12-3B2G5	Group II	*k*	IgG1	−	+	+	+
17-5C11E3	Group II	*k*	IgG2a	−	+	+	+
Q3-1C9F6	Group II	*k*	IgG1	−	+	+	+
16-5E7E8	Group II	*k*	IgG1	−	+	+	+
1-1D2B3	Group III	*k*	IgG1	+	±	+	+
8-2B4A3	Group IV	*k*	IgG1	−	±	+	+
15-4B9H10	Group V	*k*	IgA	−	±	+	+
Q4-1F10B7	Group VI	*k*	IgG1	+	+	+	+
5-1C8B6	Group VII	*k*	IgG1	−	−	+	+
Q1-1G4B2	Group VIII	*k*	IgG1	+	−	−	+
Q8-4F9F3	Group IX	*k*	IgG1	+	−	−	+

*Note:* “+” reactivity; “±” weak reactivity; “−” nonreactivity.

^a^20 MAbs were classified into nine groups based on the CLIA additivity test.

^b^ASFV/II/SC/2019 strain was used in IFA and WB.

^c^The recombinant p30 protein was expressed with a prokaryotic expression system.

## Data Availability

The data that support the findings of this study are available from the corresponding author upon reasonable request.

## Supporting Information

Supporting Information Figure S1: Expression and purification of recombinant p30 protein. Figure S2: Identification of 20 MAbs by indirect immunofluorescence assay (IFA). Figure S3: Reactivity of 20 MAbs against ASFV/II/SC/2019 was identified by WB. Figure S4: Reactivity of 20 MAbs against p30 protein was identified by WB. Figure S5: The determination of the saturation curves of the p30 antigen by each MAb in CLIA. Figure S6: Additivity index (AI) for 20 MAbs using CLIA additivity test. Figure S7: Linear regulation of the logarithmic value of the recombinant plasmid copy number and Ct value. Figure S8: The determination of the saturation curves of the p30 antigen by each MAb in ELISA. Table S1: ELISA additivity test for 20 MAbs.
