# Supplementary material for: Discovery of a Structurally Stable Immunodominant Region in ASFV p30 C-Terminus Using a Panel of Monoclonal Antibodies
Source: Transbound Emerg Dis. 2025 Nov 28;2025:8023572. doi: 10.1155/tbed/8023572 (PMC12680476; doi:10.1155/tbed/8023572)
Supplement: Supporting Information — Figure S1: Expression and purification of recombinant p30 protein. Figure S2: Identification of 20 MAbs by indirect immunofluorescence assay (IFA). Figure S3: Reactivity of 20 MAbs against ASFV/II/SC/2019 was identified by WB. Figure S4: Reactivity of 20 MAbs against p30 protein was identified by WB. Figure S5: The determination of the saturation curves of the p30 antigen by each MAb in CLIA. Figure S6: Additivity index (AI) for 20 MAbs using CLIA additivity test. Figure S7: Linear regulation of the logarithmic value of the recombinant plasmid copy number and Ct value. Figure S8: The determination of the saturation curves of the p30 antigen by each MAb in ELISA. Table S1: ELISA additivity test for 20 MAbs. [file 8023572.f1.docx]

**Discovery of a structurally stable immunodominant region in ASFV p30 C-terminus using a panel of monoclonal antibodies**

Wei Liu,^1,2^ Shandian Gao,^1,2^ Jiaoyan Su,^1^ Jinshu Sui,^1^ Tong Zhou,^1^ Jian Yang,^1^ Haiyan Lu,^1^ Huichen Guo,^1,2^ Junjun Shao,^1,2^ Huiyun Chang,^1,2^

^1^State Key Laboratory for Animal Disease Control and Prevention, Lanzhou Veterinary Research Institute, Chinese Academy of Agricultural Sciences, Lanzhou, China.

^2^Gansu Province Research Center for Basic Disciplines of Pathogen Biology, Lanzhou Veterinary Research Institute, Chinese Academy of Agricultural Sciences, Lanzhou, China

Corresponding authors: Wei Liu ([liuwei10@caas.cn](mailto:liuwei10@caas.cn)) and Junjun Shao ([shaojunjun@caas.cn](mailto:shaojunjun@caas.cn))


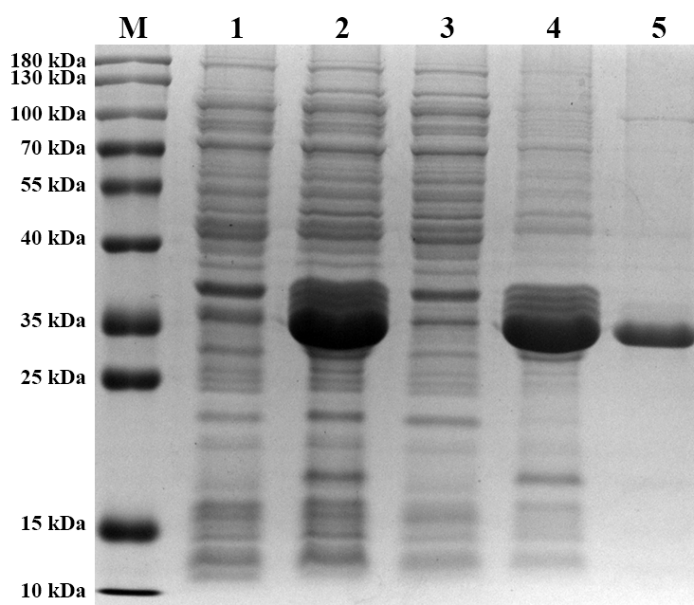


Fig. S1 Expression and purification of recombinant p30 protein. Lane M, protein ladder; lane 1, bacterial lysate before induction; lane 2, bacterial lysate after induction; lane 3, supernatant after ultrasonic lysis of bacteria; lane 4, precipitate after ultrasonic lysis of bacteria; lane 5, purified recombinant p30 protein.


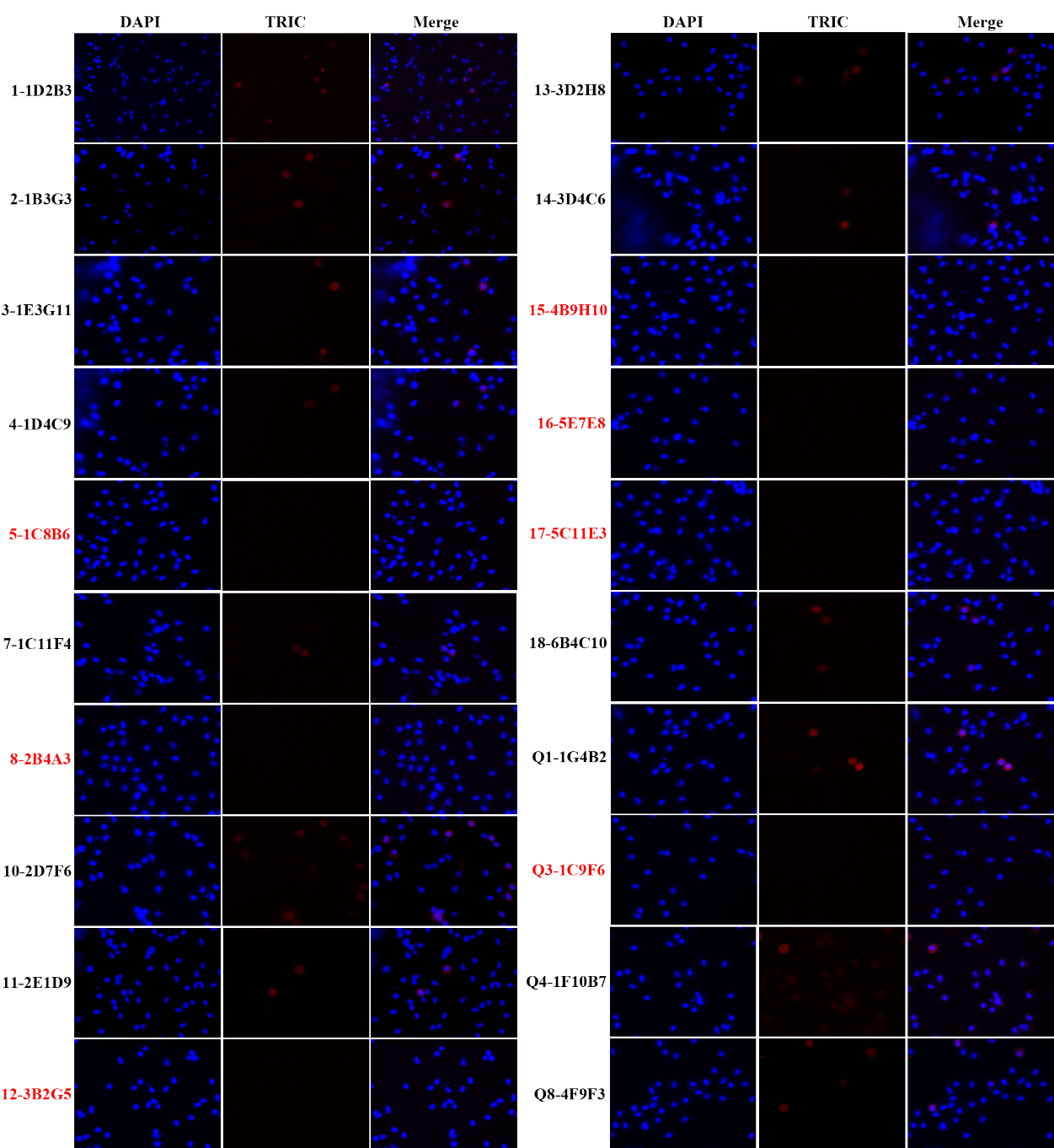


Fig. S2 Identification of 20 MAbs by indirect immunofluorescence assay (IFA). The reactivity of 20 MAbs was evaluated against ASFV/II/SC/2019-infected PAMs at multiplicity of infection (MOI) of 0.1 for 48 h.


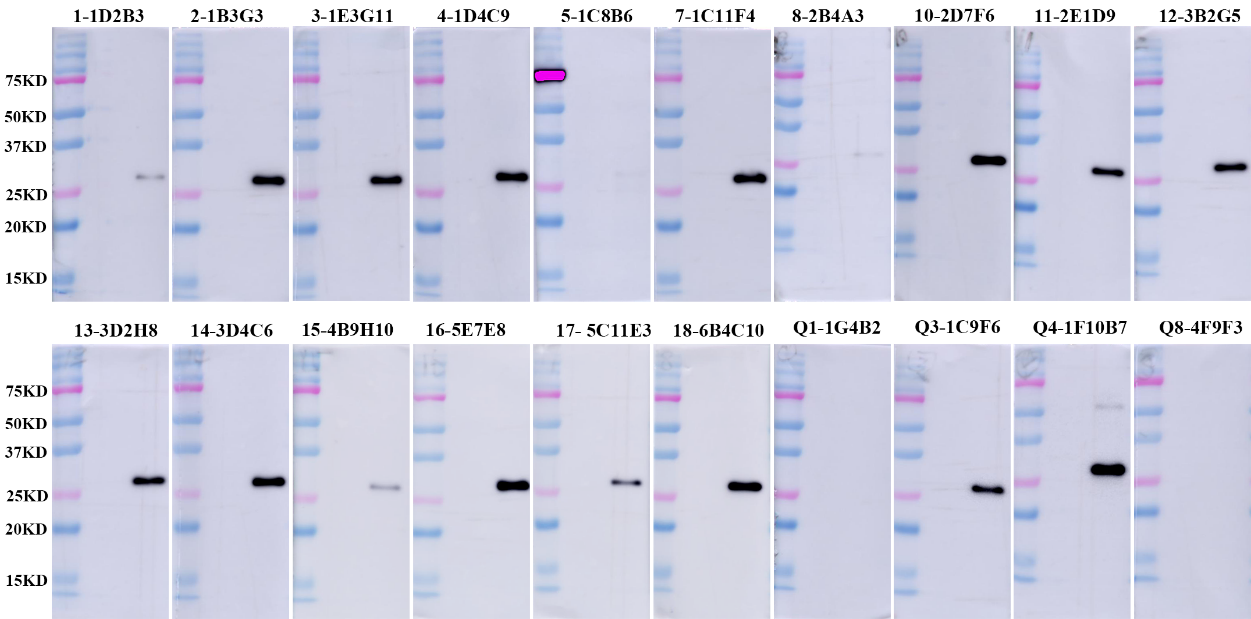


Fig. S3 Reactivity of 20 MAbs against ASFV/II/SC/2019 was identified by WB. ASFV was harvested 3 days post-infection and inactivated by incubation at 60°C for 30 min. The inactivated ASFV was then subjected electrophoresis, transferred onto polyvinylidene difluoride (PVDF) membranes, and probed with the 20 MAbs as primary antibodies.

**
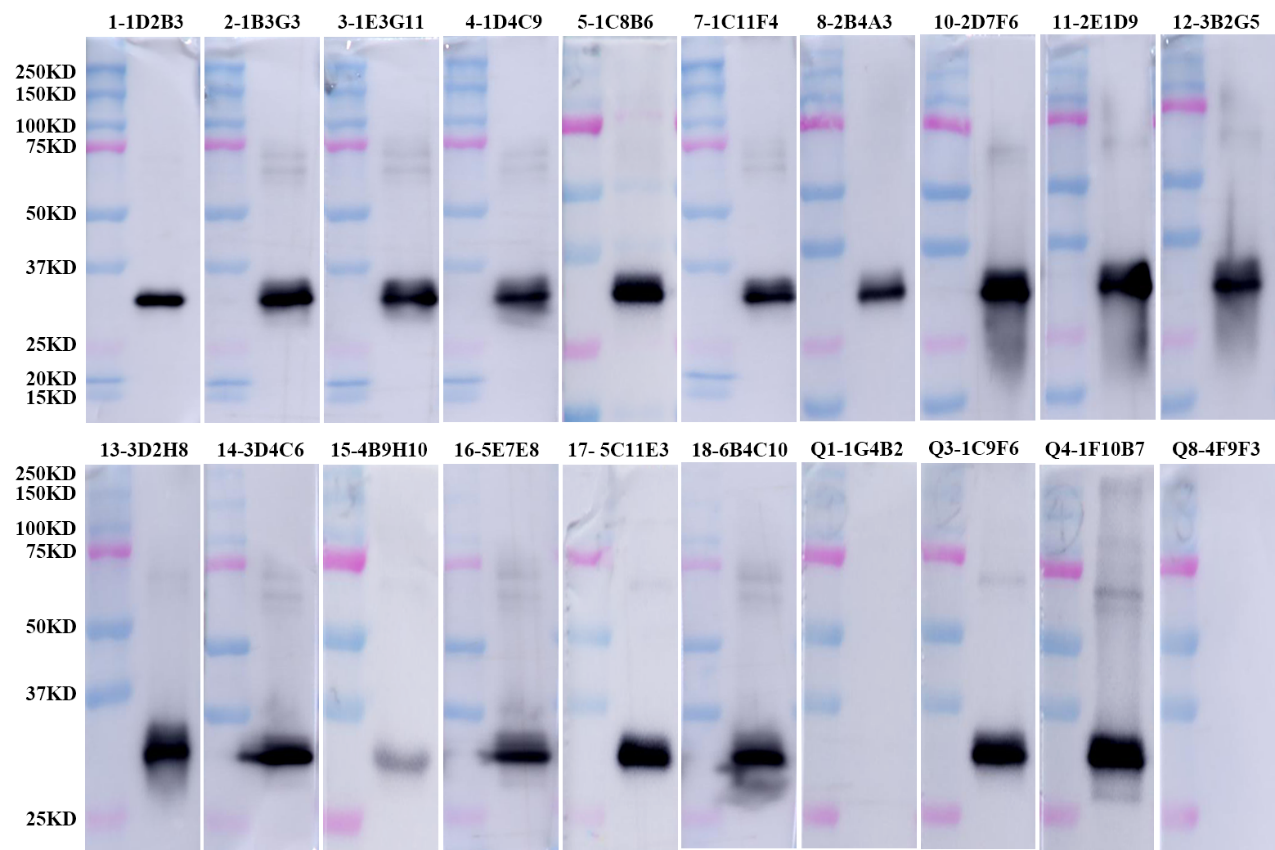
**

Fig. S4 Reactivity of 20 MAbs against p30 protein was identified by WB. p30 protein was subjected electrophoresis, transferred onto PVDF, and probed with the 20 MAbs as primary antibodies.


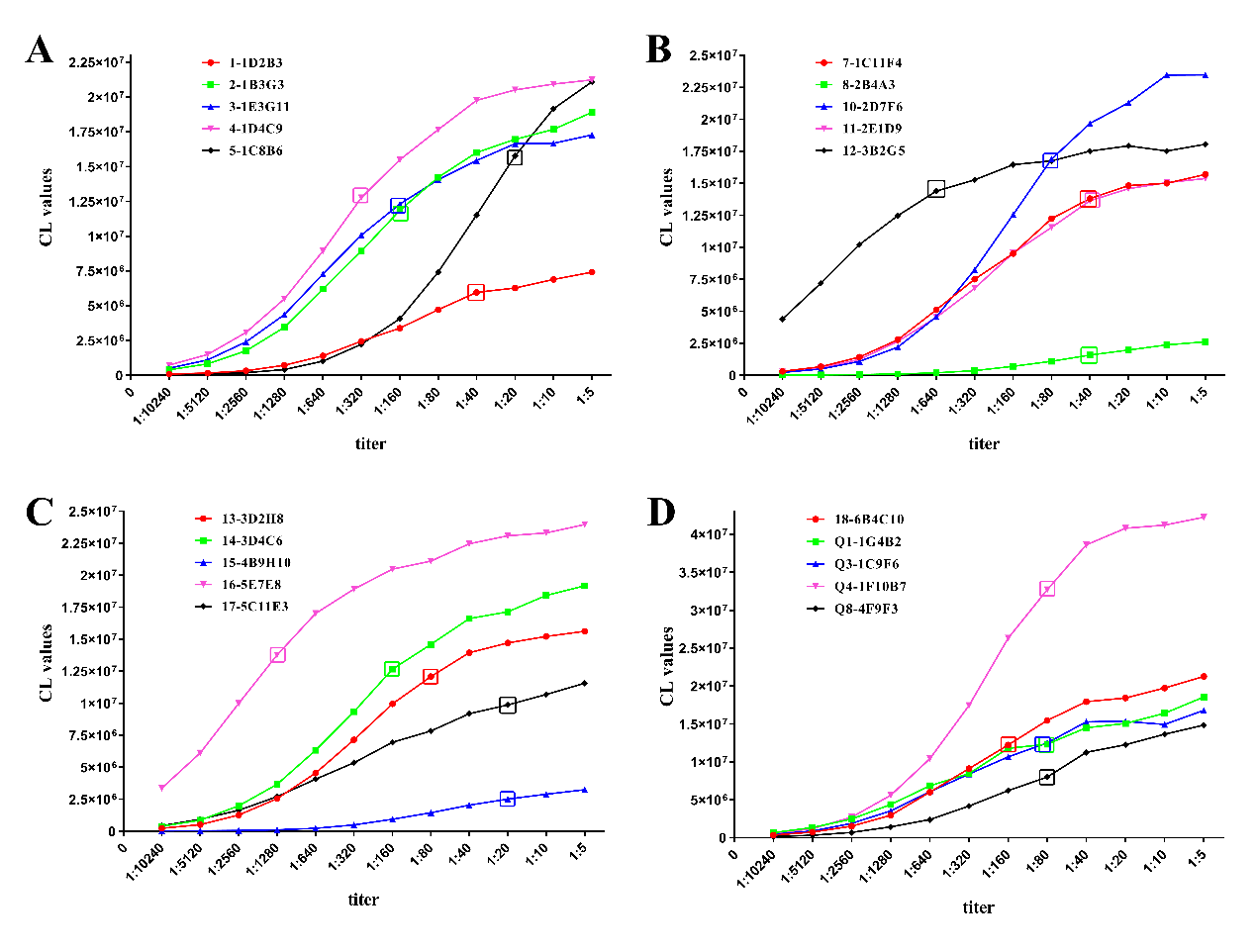


Fig. S5 The determination of the saturation curves of the p30 antigen (0.125 μg/ml in a 100 μl/well) by each MAb in CLIA. The square represents the MAbs saturation dilution, which was chosen in CLIA additivity test.


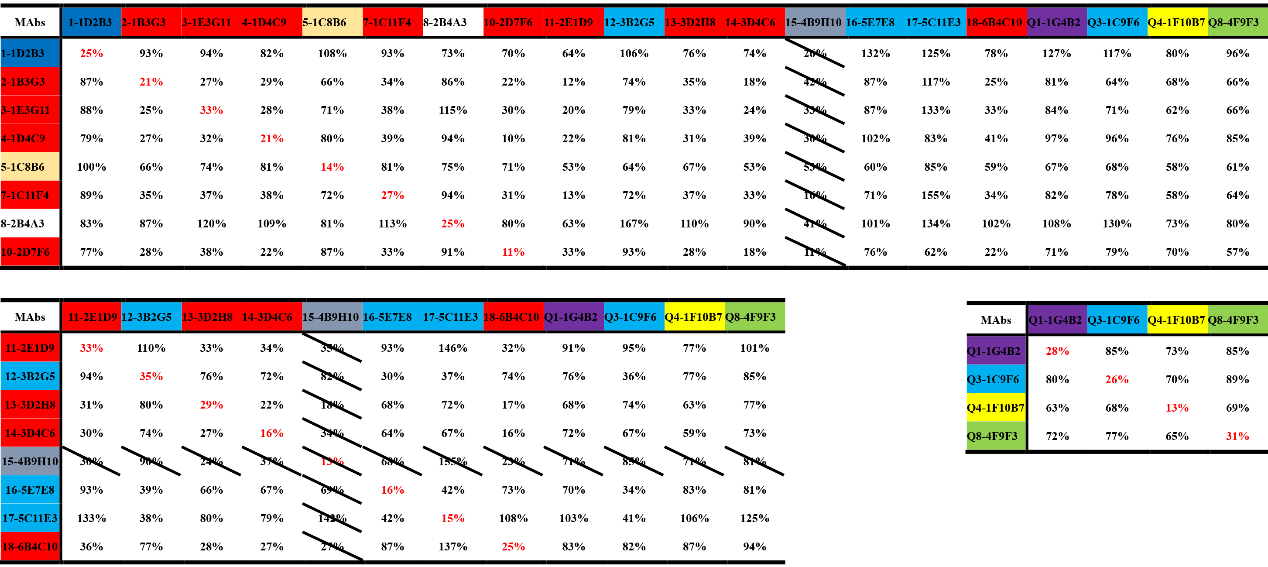


Fig. S6 Additivity index (AI) for 20 MAbs using CLIA additivity test. For each pair of MAbs, the AI higher than 50% was consider as the distinct epitopes recognized by the pair of MAbs. The same epitope was recognized by the pair of MAbs, when AI is lower than 50%.


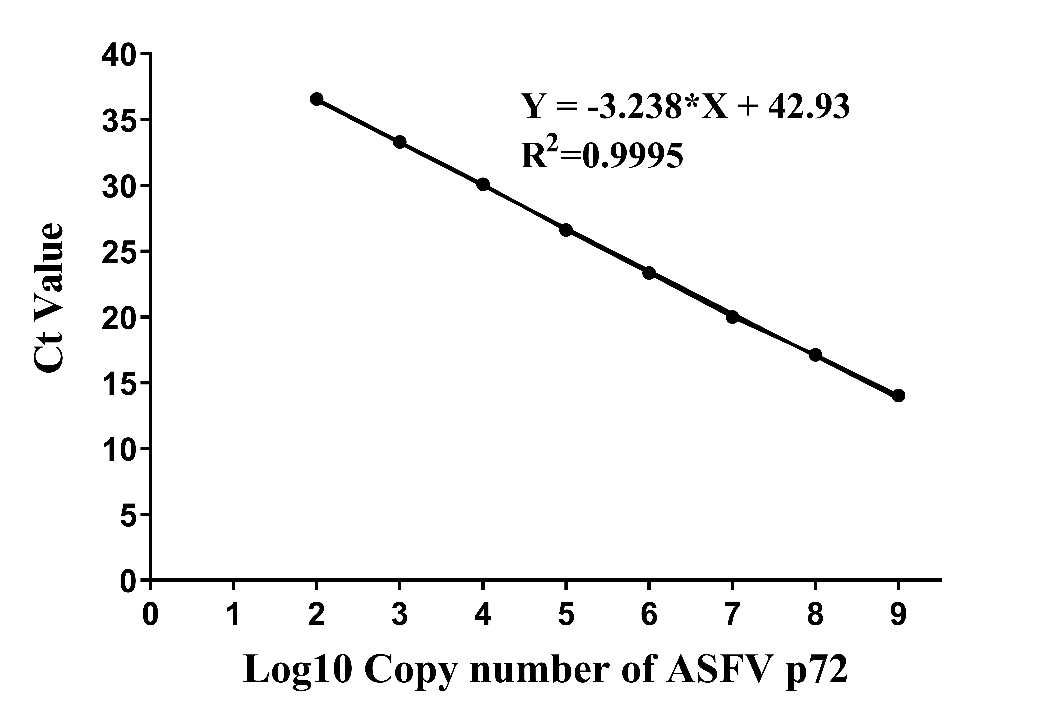


Fig. S7 Linear regulation of logarithmic value of the recombinant plasmid copy number and Ct value.


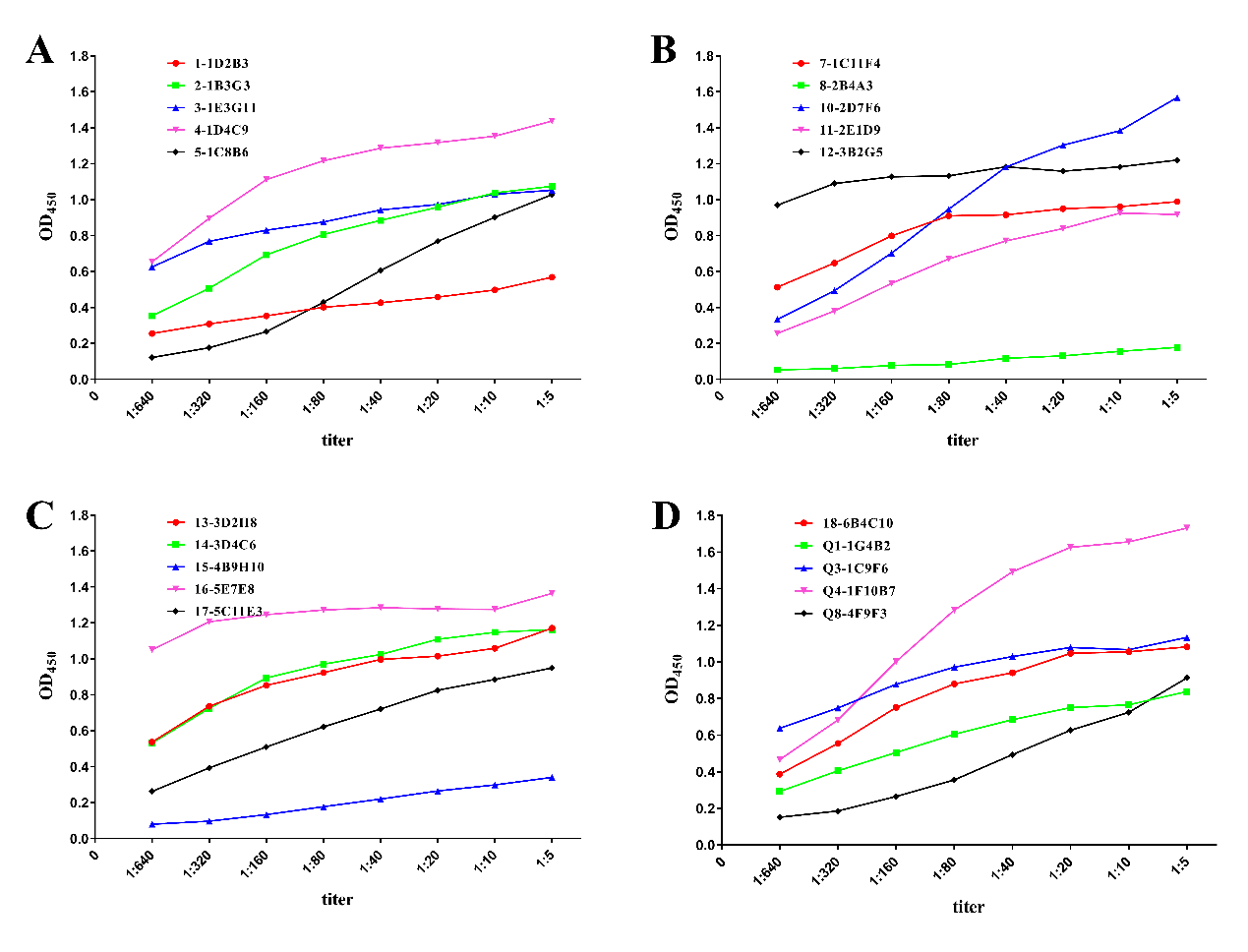
 Fig. S8 The determination of the saturation curves of the p30 antigen (0.125 μg/ml in a 100 μl/well) by each MAb in ELISA.

Table S1 ELISA additivity test for 20 MAbs.

| **MAbs** | **1-1D2B3** | **2-1B3G3** | **3-1E3G11** | **4-1D4C9** | **5-1C8B6** | **7-1C11F4** | **8-2B4A3** | **10-2D7F6** | **11-2E1D9** | **12-3B2G5** | **13-3D2H8** | **14-3D4C6** | **15-4B9H10** | **16-5E7E8** | **17-5C11E3** | **18-6B4C10** | **Q1-1G4B2** | **Q3-1C9F6** | **Q4-1F10B7** | **Q8-4F9F3** |
| --- | --- | --- | --- | --- | --- | --- | --- | --- | --- | --- | --- | --- | --- | --- | --- | --- | --- | --- | --- | --- |
| **1-1D2B3** | **-7%** | **26%** | **21%** | **27%** | **29%** | **-1%** | **30%** | **17%** | **-1%** | **63%** | **18%** | **17%** | **-10%** | **55%** | **95%** | **15%** | **44%** | **63%** | **30%** | **34%** |
| **2-1B3G3** | **23%** | **6%** | **9%** | **4%** | **28%** | **3%** | **60%** | **1%** | **1%** | **30%** | **13%** | **0%** | **25%** | **55%** | **102%** | **3%** | **44%** | **16%** | **22%** | **30%** |
| **3-1E3G11** | **12%** | **1%** | **3%** | **7%** | **25%** | **-4%** | **57%** | **4%** | **-3%** | **31%** | **12%** | **1%** | **21%** | **51%** | **99%** | **0%** | **44%** | **19%** | **31%** | **31%** |
| **4-1D4C9** | **17%** | **3%** | **12%** | **-9%** | **45%** | **15%** | **68%** | **-10%** | **9%** | **41%** | **26%** | **3%** | **9%** | **49%** | **15%** | **7%** | **68%** | **34%** | **54%** | **40%** |
| **5-1C8B6** | **25%** | **21%** | **28%** | **44%** | **-4%** | **26%** | **29%** | **45%** | **16%** | **28%** | **50%** | **29%** | **36%** | **35%** | **55%** | **33%** | **40%** | **29%** | **15%** | **21%** |
| **7-1C11F4** | **-3%** | **0%** | **0%** | **16%** | **22%** | **-5%** | **45%** | **17%** | **-7%** | **31%** | **15%** | **9%** | **-6%** | **34%** | **75%** | **10%** | **46%** | **33%** | **31%** | **30%** |
| **8-2B4A3** | **30%** | **59%** | **67%** | **70%** | **32%** | **45%** | **-7%** | **64%** | **43%** | **104%** | **66%** | **53%** | **23%** | **150%** | **209%** | **57%** | **49%** | **93%** | **40%** | **32%** |
| **10-2D7F6** | **31%** | **10%** | **21%** | **1%** | **59%** | **27%** | **87%** | **6%** | **23%** | **60%** | **18%** | **4%** | **13%** | **57%** | **19%** | **12%** | **64%** | **47%** | **55%** | **55%** |

| **MAbs** | **11-2E1D9** | **12-3B2G5** | **13-3D2H8** | **14-3D4C6** | **15-4B9H10** | **16-5E7E8** | **17-5C11E3** | **18-6B4C10** | **Q1-1G4B2** | **Q3-1C9F6** | **Q4-1F10B7** | **Q8-4F9F3** |  |  |  | **MAbs** | **Q1-1G4B2** | **Q3-1C9F6** | **Q4-1F10B7** | **Q8-4F9F3** |
| --- | --- | --- | --- | --- | --- | --- | --- | --- | --- | --- | --- | --- | --- | --- | --- | --- | --- | --- | --- | --- |
| **11-2E1D9** | **7%** | **56%** | **12%** | **15%** | **5%** | **53%** | **81%** | **4%** | **43%** | **47%** | **49%** | **54%** |  |  |  | **Q1-1G4B2** | **-1%** | **46%** | **49%** | **36%** |
| **12-3B2G5** | **45%** | **1%** | **30%** | **31%** | **50%** | **9%** | **10%** | **32%** | **46%** | **0%** | **56%** | **45%** |  |  |  | **Q3-1C9F6** | **43%** | **2%** | **44%** | **40%** |
| **13-3D2H8** | **6%** | **28%** | **-5%** | **2%** | **-10%** | **33%** | **46%** | **-1%** | **38%** | **25%** | **50%** | **49%** |  |  |  | **Q4-1F10B7** | **31%** | **36%** | **0%** | **27%** |
| **14-3D4C6** | **9%** | **24%** | **0%** | **0%** | **21%** | **41%** | **45%** | **-4%** | **37%** | **21%** | **43%** | **45%** |  |  |  | **Q8-4F9F3** | **33%** | **41%** | **33%** | **-3%** |
| **15-4B9H10** | **-3%** | **48%** | **-9%** | **23%** | **-2%** | **69%** | **54%** | **6%** | **37%** | **31%** | **50%** | **43%** |  |  |  |  |  |  |  |  |
| **16-5E7E8** | **38%** | **9%** | **36%** | **44%** | **67%** | **-1%** | **9%** | **53%** | **37%** | **1%** | **40%** | **44%** |  |  |  |  |  |  |  |  |
| **17-5C11E3** | **77%** | **10%** | **43%** | **48%** | **52%** | **15%** | **-2%** | **93%** | **71%** | **1%** | **76%** | **76%** |  |  |  |  |  |  |  |  |
| **18-6B4C10** | **4%** | **40%** | **4%** | **4%** | **14%** | **61%** | **105%** | **-3%** | **48%** | **32%** | **40%** | **39%** |  |  |  |  |  |  |  |  |
